# Automated evaluation for pericardial effusion and cardiac tamponade with echocardiographic artificial intelligence

**DOI:** 10.1093/ehjdh/ztaf112

**Published:** 2025-09-23

**Authors:** I Min Chiu, Yuki Sahashi, Milos Vukadinovic, Paul P Cheng, Susan Cheng, David Ouyang

**Affiliations:** Department of Cardiology, Smidt Heart Institute, Cedars-Sinai Medical Center, 127 S. San Vicente Blvd., Suite A3600, Los Angeles, CA 90048, USA; Department of Emergency Medicine, Chang Gung Memorial Hospital Kaohsiung Branch, Kaohsiung, Taiwan; Department of Computer Science and Engineering, National Sun Yat-sen University, Kaohsiung, Taiwan; Department of Cardiology, Smidt Heart Institute, Cedars-Sinai Medical Center, 127 S. San Vicente Blvd., Suite A3600, Los Angeles, CA 90048, USA; Department of Cardiology, Smidt Heart Institute, Cedars-Sinai Medical Center, 127 S. San Vicente Blvd., Suite A3600, Los Angeles, CA 90048, USA; Department of Bioengineering, University of California Los Angeles, Los Angeles, CA, USA; Department of Medicine, Division of Cardiology, Stanford University, Palo Alto, CA, USA; Department of Cardiology, Smidt Heart Institute, Cedars-Sinai Medical Center, 127 S. San Vicente Blvd., Suite A3600, Los Angeles, CA 90048, USA; Department of Cardiology, Smidt Heart Institute, Cedars-Sinai Medical Center, 127 S. San Vicente Blvd., Suite A3600, Los Angeles, CA 90048, USA; Division of Artificial Intelligence in Medicine, Department of Medicine, Cedars-Sinai Medical Center, Los Angeles, CA, USA

**Keywords:** Artificial intelligence, Deep learning, Pericardial effusion, Cardiac tamponade, Echocardiography

## Abstract

**Aims:**

Timely and accurate detection of pericardial effusion and assessment of cardiac tamponade remain challenging and highly operator dependent.

**Objectives:**

Artificial intelligence has advanced many echocardiographic assessments, and we aimed to develop and validate a deep learning model to automate the assessment of pericardial effusion severity and cardiac tamponade from echocardiogram videos.

**Methods and results:**

We developed a deep learning model (EchoNet-Pericardium) using temporal-spatial convolutional neural networks to automate pericardial effusion severity grading and tamponade detection from echocardiography videos. The model was trained using a retrospective dataset of 1 427 660 videos from 85 380 echocardiograms at Cedars-Sinai Medical Centre (CSMC) to predict PE severity and cardiac tamponade across individual echocardiographic views and an ensemble approach combining predictions from five standard views. External validation was performed on 33 310 videos from 1806 echocardiograms from Stanford Healthcare (SHC). In the held-out CSMC test set, EchoNet-Pericardium achieved an AUC of 0.900 (95% CI: 0.884–0.916) for detecting moderate or larger pericardial effusion, 0.942 (95% CI: 0.917–0.964) for large pericardial effusion, and 0.955 (95% CI: 0.939–0.968) for cardiac tamponade. In the SHC external validation cohort, the model achieved AUCs of 0.869 (95% CI: 0.794–0.933) for moderate or larger pericardial effusion, 0.959 (95% CI: 0.945–0.972) for large pericardial effusion, and 0.966 (95% CI: 0.906–0.995) for cardiac tamponade. Subgroup analysis demonstrated consistent performance across ages, sexes, left ventricular ejection fraction, and atrial fibrillation statuses.

**Conclusion:**

Our deep learning-based framework accurately grades pericardial effusion severity and detects cardiac tamponade from echocardiograms, demonstrating consistent performance and generalizability across different cohorts. This automated tool has the potential to enhance clinical decision-making by reducing operator dependence and expediting diagnosis.

## Graphical Abstract

**Graphical Abstract:**
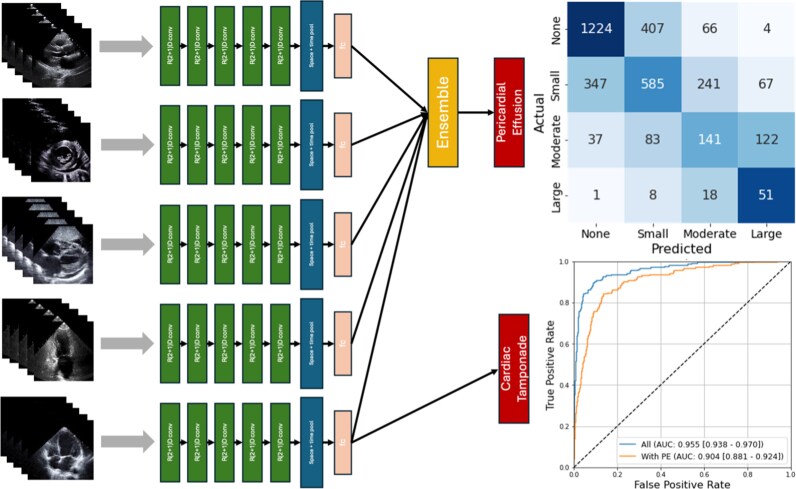
EchoNet-Pericardium: A temporal-spatial convolutional neural network designed to automate pericardial effusion severity grading and tamponade detection using echocardiography videos. The model integrates predictions from five standard echocardiographic views through an ensemble approach, achieving an AUC of 0.900 for detecting moderate or larger pericardial effusion, 0.942 for large pericardial effusion, and 0.955 for cardiac tamponade.

## Introduction

Cardiac tamponade, a life-threatening condition, occurs when excess fluid accumulates in the pericardial space, resulting in increased intrapericardial pressure, impairs cardiac filling, and reduces cardiac output. Without prompt recognition and intervention, this can lead to shock and circulatory collapse. Echocardiography is the gold standard for pericardial effusion detection due to its accessibility, portability, and comprehensive assessment of both anatomy and function^1–3^ However, despite increasing availability of ultrasound technology, the accurate assessment of pericardial effusion and cardiac tamponade still depends on expert image acquisition and interpretation. Distinguishing the severity of pericardial effusion and presence of tamponade can be challenging, with mild effusions sometimes mistaken for pericardial fat, and more critically, the severity of effusion does not necessarily correlate with the risk of tamponade.^4^

These challenges highlight the need for a reliable, automated method for detecting and assessing pericardial effusions that minimizes operator dependence and effectively assesses the risk of tamponade. Artificial intelligence (AI) has shown significant success in evaluating echocardiography tasks, including the assessment of left ventricular function, wall motion abnormalities, right ventricular function, and valvular disease^5–9^ AI presents a promising approach to automating complex image interpretation, improving the precision of measurements, and identify subtle cardiac phenotypes. While previous studies assessing pericardial effusions with deep learning have been limited by small training datasets and lack of eternal validation,^10,11^ such that further work is warranted to apply AI to assessing pericardial effusion severity and predicting progression to cardiac tamponade.

In this study, we developed and evaluated performance of a deep learning pipeline in automating identification of PE and cardiac tamponade from standard transthoracic echocardiogram studies. We hypothesized that a deep learning approach can identify and assess PE severity on combinations of standard echocardiogram view videos with high-throughput automation.

## Methods

### Study populations and data collection

In this study, we analyzed previously collected transthoracic echocardiogram studies from Cedars-Sinai Medical Centre (CSMC) between 12 September 2011, and 4 June 2022. Echocardiogram videos were originally stored as DICOM videos from GE or Philips ultrasound machines. We pre-processed these videos to remove non-ultrasound sector information, extracted metadata, and converted them to AVI format.^12^ Videos were stored as 112 × 112-pixel video files and view classified into five standard echocardiographic views (apical-4-chamber, apical-2-chamber, parasternal long axis, parasternal short axis, and subcostal views). The view classifier model used in this study was trained on 77 426 echocardiogram videos to classify 58 specific view categories. Pericardial effusion severity and presence of cardiac tamponade for was extracted from the clinical report determined in a high-volume echocardiography laboratory in accordance with American Society of Echocardiography guidelines.^13^ Cardiac tamponade was determined by board-certified cardiologists based on echocardiographic assessment, with criteria included right atrial or right ventricular diastolic collapse, plethoric inferior vena cava with blunted respiratory variation, and evidence of ventricular interdependence. Label extraction was confirmed via structured report parsing. The model was trained on four classes of pericardial effusion sizes (none, mild, moderate, and large), with intermediate categories were assigned to the more severe category.

EchoNet-Pericardium—a full end-to-end approach including video processing, view classification, pericardial effusion size assessment, and identification of cardiac tamponade—was externally validated using data from an independent and geographically distinct high-volume echocardiography lab. The model was evaluated on 1806 studies (containing a total of 33 310 videos) from the Stanford Healthcare (SHC). The ensemble prediction utilized predictions from five standard echocardiographic views for evaluating pericardial effusion size and tamponade model validation. Model output was compared with clinical grading determined by expert cardiologists from the clinical reports. This study was approved by the institutional review boards at CSMC and SHC.

### AI model

Echocardiogram videos were divided into training, validation, and test datasets in an 8:1:1 ratio by patient, ensuring no patient overlap across the sets to prevent data leakage. If a patient had multiple echocardiogram studies or videos, each video was treated as an independent example during model training. Deep learning models were trained using the PyTorch Lightning deep learning framework. Video-based convolutional neural networks (R2 + 1D) were used for PE severity assessment and cardiac tamponade prediction.^14^ This model architecture was previously used for other echocardiography tasks and shown to be effective.^6,9^ The models were initialized with random weights and trained using a binary cross entropy loss function for up to 50 epochs, using an ADAM optimizer, an initial learning rate of 1e−2, and a batch size of 64 on NVIDIA RTX A6000 GPU. Early stopping was performed based on the validation loss if no improvement after 5 epochs. For model training, we selected random clips of 32 frames, sampled every other frame to obtain 16 frames per clip, and input these into the model. For pericardial effusion size assessment, models were trained for each individual view, with input videos of each individual view, and an ensemble model was constructed by logistical regression with inputs of the inference prediction from models of each view. For tamponade prediction, we trained the model using all apical-4-chamber videos for binary prediction.

### Statistical analysis

Continuous variables are expressed as mean ± standard deviation, while nominal variables are presented as proportions. All analyses were performed on the held-out test dataset and external test set, which was never seen during model training. Model performance was evaluated using area under the receiver operating characteristic curve and confusion matrices. Confidence intervals were computed using 1000 bootstrapped samples of the test datasets. Statistical analysis was performed in Python (version 3.8.0). Subgroup analysis was conducted to assess model performance in patients with different age, sex, race, and other clinical characteristics. Clinical characteristics were obtained from the electronic health record or associated echocardiography report.

## Results

### Primary cohort characteristics

A total of 85 380 transthoracic echocardiograms from 49 598 patients at CSMC, collected between 12 September 2011, and 4 June 2022, were used to train and evaluate the deep learning models. The mean (SD) age of the patients was 68.7 (19.4) years, with 49.7% being male. Among them, 3.5% had atrial fibrillation, 12.2% had heart failure, 44.9% had hypertension, and 19.4% had diabetes mellitus. Across all echocardiograms, 14.0% showed small pericardial effusion, 4.2% had moderate pericardial effusion, 1.1% had large pericardial effusion, and 0.7% presented with cardiac tamponade (*Table 1*). Echocardiograms were divided into training, validation, and test datasets in an 8:1:1 ratio by patient, resulting in 68 110 echocardiograms in the training set, 8508 in the validation set, and 8762 in the test set. A total of 1 427 660 videos were included through the view classifier process, with 363 817 from A4C, 179 361 from A2C, 355 330 from PLAX, 294 950 from PSAX, and 234 202 from Subcostal view (see Supplementary material online, *Table S1*).

**Table 1:** Patient characteristics

	CSMC			
Characteristic	Train	Validation	Test	SHC
Number of patients	39 654	4934	5010	1806
Sex (% Male)	19 711 (49.7)	2438 (49.4)	2 490 (49.7)	957 (53.0)
Age, mean (std), y	68.7 (19.4)	68.5 (19.3)	68.6 (19.3)	60.4 (17.3)
Race				
Caucasian, *n* (%)	26 944 (67.9%)	3393 (68.8%)	3419 (68.2%)	907 (50.2)
Black, *n* (%)	5774 (14.6%)	717 (14.5%)	734 (14.7%)	76 (4.2)
Asian, *n* (%)	2981 (7.5%)	343 (7.0%)	364 (7.3%)	303 (16.8)
Others, *n* (%)	3955 (10.0%)	481 (9.7%)	493 (9.8%)	163 (9.0)
Clinical History				
Atrial Fibrillation	1375 (3.5%)	179 (3.6%)	190 (3.8%)	542 (30.0)
Heart Failure	4828 (12.2%)	616 (12.5%)	602 (12.0%)	751 (41.6)
CAD	8796 (22.2%)	1138 (23.1%)	1094 (21.8%)	298 (16.5)
Hypertension	17 811 (44.9%)	2221 (45.0%)	2 262 (45.1%)	1082 (59.9)
Diabetes Mellitus	7679 (19.4%)	994 (20.1%)	954 (19.0%)	168 (9.3)
Number of Study	68 110	8508	8762	1806
Number of Video	1 139 533	142 341	145 786	33 310
Pericardial Effusion				
Small	9512 (14.0%)	1193 (13.6%)	1242 (14.2%)	83 (4.6%)
Moderate	2831 (4.2%)	336 (3.9%)	383 (4.4%)	19 (1.1%)
Large	796 (1.2%)	77 (0.9%)	78 (0.9%)	11 (0.6%)
Cardiac Tamponade	482 (0.7%)	64 (0.8%)	39 (0.4%)	17 (0.9%)

### Model performance in the primary cohort

**Figure 1:**
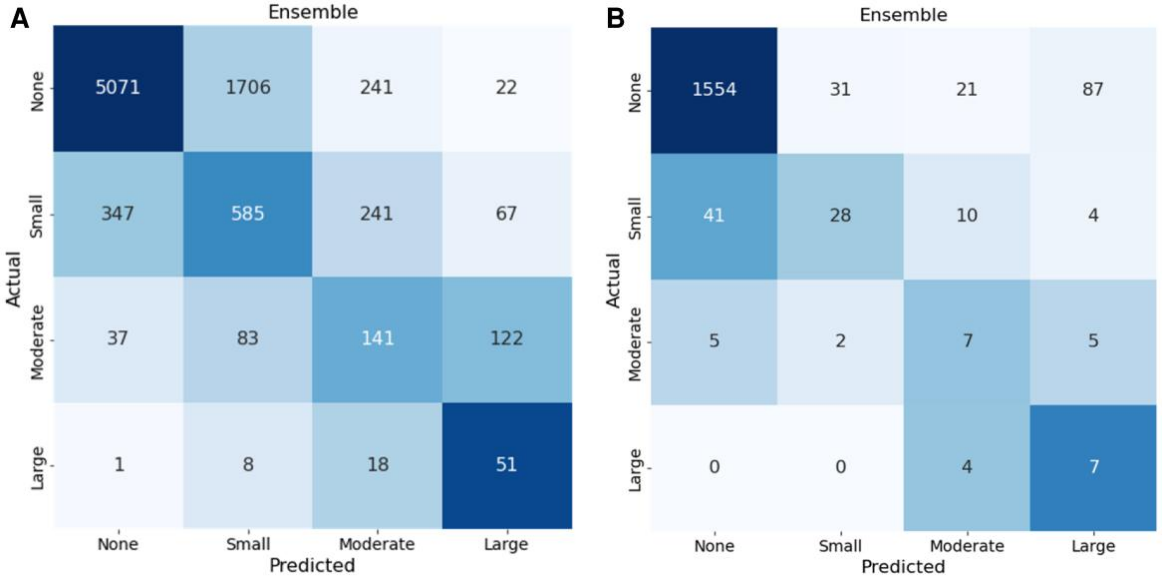
Confusion matrices for pericardial effusion severity classification in the primary cohort (*A*) and the external validation cohort (*B*). The EchoNet-Pericardium model utilizes an ensemble of predictions from five standard echocardiographic views to classify pericardial effusion as none, small, moderate, or large. *Figure 1A* demonstrates performance in the primary test cohort, while *Figure 1B* depicts results in the external validation cohort. The diagonal values indicate correct predictions, with the model showing consistent accuracy across different levels of effusion severity.

We trained five view-specific deep learning models and ensembled them to obtain the final pericardial effusion size assessment (*Table 2*). Our ensemble model effectively distinguished PE severity, achieving an AUC of 0.900 (0.884–0.916) for detecting moderate or above PE and 0.942 (0.917–0.964) for large PE in the primary cohort. As shown in the confusion matrix (*Figure 1A*), the model performance in the test set of the primary cohort for detecting moderate or larger pericardial effusion demonstrated a sensitivity of 0.72 (0.698–0.741), a specificity of 0.931 (0.913–0.949), and a negative predictive value (NPV) of 0.984 (0.969–0.999). For detecting large pericardial effusions, the model achieved a sensitivity of 0.654 (0.622–0.686), a specificity of 0.976 (0.958–0.994), and an NPV of 0.997 (0.981–1.0). These metrics indicate the model’s accuracy in identifying and excluding cases of moderate or severe PE and large PE.

**Table 2:** EchoNet-pericardium model performance

Characteristic	Small PE	Moderate PE	Large PE
CSMC			
Apical 4 Chamber	0.779 (0.766–0.792)	0.890 (0.875–0.904)	0.944 (0.918–0.965)
Apical 2 Chamber	0.734 (0.720–0.747)	0.841 (0.818–0.862)	0.872 (0.815–0.926)
Parasternal long axis	0.645 (0.633–0.658)	0.745 (0.721–0.770)	0.837 (0.785–0.880)
Parasternal short axis	0.710 (0.697–0.723)	0.810 (0.788–0.832)	0.887 (0.842–0.924)
Subcostal	0.630 (0.617–0.643)	0.722 (0.696–0.747)	0.761 (0.701–0.819)
Ensemble of all views	0.805 (0.792–0.816)	0.900 (0.884–0.916)	0.942 (0.917–0.964)
SHC			
Ensemble of all views	0.746 (0.697–0.790)	0.869 (0.794–0.933)	0.959 (0.945–0.972)

**Figure 2:**
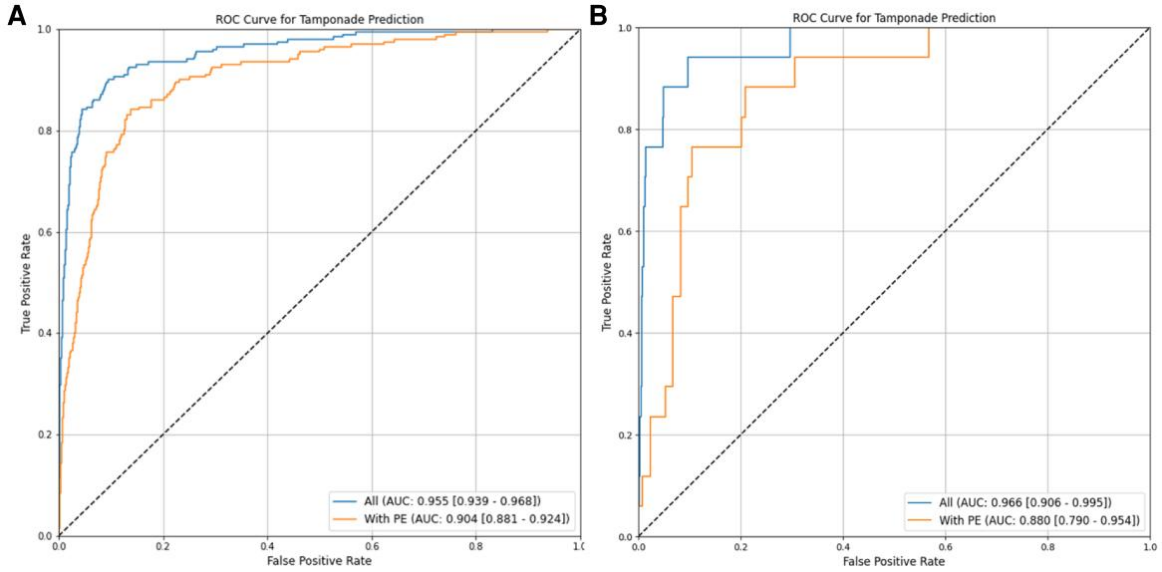
Receiver operating characteristic curves for cardiac tamponade prediction in the primary cohort (*A*) and the external validation cohort (*B*). The EchoNet-Pericardium model distinguishes cardiac tamponade cases using apical-4-chamber views. The blue curves represent model performance on all echocardiograms, while the orange curves show performance specifically for studies with pericardial effusion. *Figure 2A* corresponds to the primary test cohort, achieving an AUC of 0.955 for all echocardiograms and 0.904 for cases with effusion. *Figure 2B* represents the external validation cohort, with an AUC of 0.966 for all echocardiograms and 0.880 for cases with effusion.

For cardiac tamponade, the model, trained using A4C views, achieved an AUC of 0.955 (0.939–0.968). When evaluated on echocardiogram studies with pericardial effusion, the model distinguished cases of tamponade with an AUC of 0.904 (0.881–0.924) (*Figure 2A*). When restricted to echocardiograms with pericardial effusion, the model achieved a sensitivity of 0.870 and specificity of 0.842 for cardiac tamponade prediction, with a positive predictive value of 0.157 and NPV of 0.995 (*Figure 2A*). This reflects the model’s ability to identify tamponade physiology specifically among patients with detected effusions.

### Subgroup analysis

In the subgroup analysis, the model demonstrated consistent performance in predicting moderate or larger pericardial effusion and cardiac tamponade across various subgroups within the test set (*Table 3*). For patients over 65 years, the AUC for moderate or larger pericardial effusion was 0.901 (95% CI: 0.882–0.921), and for tamponade, it was 0.953 (95% CI: 0.934–0.970). Performance across gender was similar, with an AUC of 0.896 (95% CI: 0.872–0.918) in males and 0.902 (95% CI: 0.881–0.923) in females for moderate or larger pericardial effusion, and 0.974 (95% CI: 0.960–0.984) in males and 0.944 (95% CI: 0.921–0.964) in females for tamponade.

**Table 3:** Subgroup analysis

Characteristic	Case	Moderate PE or worse	Tamponade
All	8762	0.900 (0.884–0.916)	0.955 (0.939–0.969)
Age > 65 years old	5319	0.901 (0.882–0.921)	0.953 (0.934–0.970)
Age < 65 years old	3443	0.897 (0.868–0.922)	0.955 (0.925–0.977)
Male	4388	0.896 (0.872–0.918)	0.974 (0.960–0.984)
Female	4374	0.902 (0.881–0.923)	0.944 (0.921–0.964)
Caucasian	5955	0.891 (0.870–0.911)	0.959 (0.942–0.972)
Black	1323	0.899 (0.864–0.931)	0.936 (0.892–0.975)
Asian	668	0.951 (0.925–0.972)	—
LVEF > 50	6875	0.908 (0.891–0.925)	0.970 (0.956–0.982)
LVEF < 50	1688	0.898 (0.855–0.930)	0.900 (0.848–0.942)
Atrial fibrillation during examination	378	0.901 (0.831–0.951)	0.979 (0.969–0.988)
Without Atrial fibrillation during examination	8384	0.900 (0.882–0.915)	0.955 (0.939–0.970)

The model’s performance was further evaluated across different ranges of left ventricular ejection fraction (LVEF). In patients with LVEF below 50, the model achieved AUCs of 0.898 (95% CI: 0.855–0.930) for moderate or larger pericardial effusion and 0.899 (95% CI: 0.848–0.942) for tamponade. Among patients with atrial fibrillation during examination, the model achieved an AUC of 0.901 (95% CI: 0.831–0.951) for moderate or larger pericardial effusion and 0.979 (95% CI: 0.969–0.988) for tamponade, while in patients without atrial fibrillation, the AUCs were 0.900 (95% CI: 0.882–0.915) and 0.955 (95% CI: 0.939–0.970) for moderate or larger pericardial effusion and tamponade, respectively.

### External validation

The external validation cohort from SHC consisted of 1806 patients with a mean (SD) age of 60.4 (17.3) years, and 53.0% of them were male. Regarding race, 50.2% of the patients were Caucasian, 4.2% were Black, and 16.8% were Asian. Clinical history data revealed that 30.0% of the patients had atrial fibrillation, 41.6% had heart failure, 16.5% had CAD, 59.9% had hypertension, and 9.3% had diabetes mellitus. The dataset included 1806 echocardiograms, corresponding to 33 310 videos. Among these, 4.6% showed small pericardial effusion, 1.1% had moderate pericardial effusion, 0.6% had large pericardial effusion, and 0.9% of cases presented with cardiac tamponade. A total of 33 310 videos were included through the view classifier process, with 9462 from A4C, 4040 from A2C, 7982 from PLAX, 6601 from PSAX, and 5225 from Subcostal view (see Supplementary material online, *Table S2*).

The EchoNet-Pericardium showed consistent performance in distinguishing pericardial effusion size and tamponade in the external validation cohort. The model demonstrated an AUC of 0.869 (0.794–0.933) in detecting at least moderate pericardial effusion and an AUC of 0.959 (0.945–0.972) for large pericardial effusion. As shown in the confusion matrix (*Figure 1B*), the model’s performance in the external validation cohort for detecting moderate or larger pericardial effusion demonstrated a sensitivity of 0.737 (0.539–0.935), a specificity of 0.976 (0.968–0.983), and an NPV of 0.997 (0.994–1.0). For detecting large pericardial effusions, the model achieved a sensitivity of 0.636 (0.352–0.921), a specificity of 0.943 (0.932–0.954), and an NPV of 0.997 (0.995–1.0).

For cardiac tamponade, the model achieved an AUC of 0.966 (0.906–0.995). When evaluated on echocardiograms with at least a small pericardial effusion, the model distinguished cases of tamponade with an AUC of 0.880 (0.790–0.954) (*Figure 2B*). When restricted to echocardiograms with pericardial effusion, the model achieved a sensitivity of 0.891 and specificity of 0.792 for tamponade prediction, with a positive predictive value of 0.431 and NPV of 0.974.

## Discussion

This study presents a validated deep learning framework capable of accurately assessing PE severity and detecting cardiac tamponade using echocardiography. From a full transthoracic echocardiogram, EchoNet-Pericardium effectively identified pericardial effusion size from five standard echocardiographic views, achieving an AUC of 0.900 for moderate or larger pericardial effusion and 0.942 for large PE in primary cohorts. Our model also demonstrated high accuracy in detecting cardiac tamponade with an AUC of 0.955. EchoNet-Pericardium demonstrated consistent performance across a geographically distinct external validation cohort without preselection, showcasing its robustness and generalizability in real-world settings. Implementing this automated tool may support timely diagnosis, reduce operator dependence, and have the potential to improve clinical workflow (*Graphical Abstract*).

For a comprehensive clinical evaluation of pericardial effusions and cardiac tamponade, clinicians use a variety of echocardiographic views are assessed together. In EchoNet-Pericardium, the use of video-based convolutional neural networks (R2 + 1D) trained for each view and then ensembled captures temporal dynamics crucial for identifying pericardial effusion size and distinguishing cases with tamponade. For classifying tamponade, we chose the A4C view to focus on given the appearance of all four chambers that allows the model to assess intraventricular dependence and associations between the left and right heart. Our study builds on the intersection of AI and echocardiography, advancing previous achievements in cardiovascular imaging applications. Significant progress has been made in automating view classification,^15,16^ identifying structural abnormalities like left ventricular hypertrophy,^8,17^ evaluating left ventricular systolic function,^6^ and assessing valvular function and vascular emergency.^9,18,19^ By integrating our model with existing AI-driven innovations, we aim to support less experienced operators in obtaining high-quality imaging,^20,21^ thereby improving diagnostic accuracy for pericardial effusion and enabling early detection of tamponade.

Future research could focus on refining automated quantification, validating model performance across varied clinical settings, and expanding access to diagnostic tools in primary care and resource-limited regions. In parallel, clinical integration will be essential. At present, the model is designed for retrospective analysis of stored echocardiography videos, with future applications envisioned in *post hoc* reporting and automated triage through integration with institutional imaging and reporting systems. Embedding the model into reporting workflows could streamline interpretation and flag high-risk studies for expedited review. While a standalone user-interface prototype has not yet been developed, advancing these integration pathways will be crucial for clinical deployment. An additional future direction will be the incorporation of interpretability strategies, such as saliency mapping or attention mechanisms, to enhance clinician trust, support regulatory review, and provide insights into model decision-making.

This study has certain limitations that should be considered when interpreting the results. First, our labels were derived from expert echocardiography reports, which may introduce biases inherent in subjective interpretation. Although our large dataset and high-volume echo lab reduce inter-reader variability, further work with multi-reader adjudication or consensus standards could strengthen label reliability. Second, although trained on a large dataset, the model has not yet been evaluated in real-time clinical practice, particularly in point-of-care ultrasonography by non-cardiologists. These settings often involve variable image quality due to suboptimal acoustic windows or off-axis imaging, and operator experience that may differ from that of cardiologists.^22^ While our model demonstrated robustness in a large external cohort, future studies should assess its performance under these practical conditions, including low-quality bedside acquisitions. To address this, we plan prospective studies of EchoNet-Pericardium in point-of-care settings with diverse users to assess model performance under suboptimal conditions and its impact on diagnostic confidence, workflow efficiency, and downstream clinical decision-making. While our cross-institution results indicate robustness to practice variation, further prospective validation is needed to confirm generalizability and real-world clinical benefit.

In conclusion, our study presents a novel, automated approach for detecting and grading pericardial effusion severity and identifying cardiac tamponade using deep learning models applied to echocardiography. EchoNet-Pericardium’s consistent performance across multiple datasets highlights its potential to support clinical decision-making and streamline echocardiographic assessment of PE and cardiac tamponade. As an assistive decision-support tool, it functions as a triage aid that reduces manual effort by automatically grading effusion severity and flagging features suggestive of tamponade. This standardization could help reduce inter-operator variability and enhance diagnostic confidence in both routine and emergency settings. Prospective studies and integration into clinical practice will be essential to fully realize the benefits of this technology.

## Supplementary Material

ztaf112_Supplementary_Data

## Data Availability

The data underlying this article cannot be shared publicly due to the privacy of the individuals who participated in the study. The code and trained model weights are available at https://github.com/echonet/pericardium.
